# MPO density in primary cancer biopsies of ovarian carcinoma enhances the indicative value of IL-17 for chemosensitivity

**DOI:** 10.1186/s12885-016-2673-7

**Published:** 2016-08-17

**Authors:** Raoul A. Droeser, Robert Mechera, Silvio Däster, Benjamin Weixler, Marko Kraljević, Tarik Delko, Uwe Güth, Sylvia Stadlmann, Luigi Terracciano, Gad Singer

**Affiliations:** 1 Department of Surgery, University Hospital Basel, Spitalstrasse 21, 4031 Basel, Switzerland; 2 Institute for Surgical Research and Hospital Management ICFS, Hebelstrasse 20, 4031 Basel, Switzerland; 3 Department of Gynecology and Obstetrics, Kantonsspital Winterthur, Brauerstrasse 15, 8400 Winterthur, Switzerland; 4 Department of Gynecology and Obstetrics, University Hospital Basel, Spitalstrasse 21, 4031 Basel, Switzerland; 5 Institute of Pathology, Kantonsspital Baden AG, Im Ergel 1, 5404 Baden, Switzerland; 6 Institute of Pathology, University Hospital Basel, Schönbeinstrasse 40, 4031 Basel, Switzerland

**Keywords:** Myeloperoxidase, Interleukin-17, Synergistic effect, Ovarian cancer, Chemosensitivity

## Abstract

**Background:**

Cancer of the ovary is mostly discovered at a late stage and cannot be removed by surgery alone. Therefore surgery is usually followed by adjuvant chemotherapy. However, few reliable biomarkers exist to predict response to chemotherapy of ovarian cancer. Previously, we could demonstrate that IL-17 density is indicative for chemosensitivity. This study focuses on the predictive value of myeloperoxidase (MPO) concerning response to chemotherapy of ovarian cancer.

**Methods:**

Biopsies of mostly high-grade primary serous ovarian carcinomas and their matched recurrences were stained with MPO after fixation in formalin and embedding in paraffin. For this staining the technique of tissue-microarray was used. Recurrence within 6 months of the completion of platinum-based chemotherapy was defined as chemoresistance as previously publised. Data for MPO could be analyzed in 92 biopsies.

**Results:**

MPO and IL-17 positive immune cells correlated significantly in biopsies of primary and recurrent carcinomas (*r*_s_ = 0.41; *p* = 0.004 and *r*_s_ = 0.40; *p* = 0.007, respectively). MPO expression alone did not predict response to chemotherapy, but in multivariate cox regression analysis including age, residual disease, number of chemotherapy cycles, FIGO classification and combined categorized MPO and IL-17 cell densities of primary cancer biopsies, the combination of both immune markers was an independent prognostic factor for recurrence-free survival (*p* = 0.013, HR = .23, 95CI = 0.07–0.73). There was no chemoresistant patient in the subgroup of MPO + IL-17+, neither in primary nor in recurrent cancer biopsies.

**Conclusions:**

High MPO positive cell density enhances the indicative value of IL-17 for response to chemotherapy in ovarian carcinoma. Although, these results have to be validated in a larger cohort.

## Background

Ovarian cancer has an incidence range of 5-15/100’000 in Europe [1–3]. It is only the 5^th^ most common female cancer, but even though it is the most lethal of all female genital carcinomas. It is mostly discovered at a late stage and cannot be removed by surgery alone due to late and unspecific symptoms. Surgical debulking is usually followed by adjuvant platinum-based chemotherapy. However, most patients recur with chemoresistant disease.

It is known that tumor microenvironment influences tumor biology and that tumor behavior is affected by the immunological environment. According to several previous publications tumor microenvironment seems to have significant impact on survival and tumor growth [4–8]. Tumor-infiltrating lymphocytes (TILs) are frequently thought to mirror tumor immune response to invasive neoplasms [9]. They were discovered in different solid tumors [10, 11]. Indeed, in colorectal cancer (CRC) the “immune contexture” [12], especially cells of the adaptive immune response, are more important concerning prognosis than the TNM staging and might help in decision making for personalized treatment [13, 14]. There are several previous studies that analyzed different markers predicting response to platinum-based chemotherapy in ovarian cancer with the scope to optimize adjuvant treatment [15–17]. However, only few markers were helpful.

On one side it is known that FOXP3 positive regulatory T (Treg) cell infiltration is associated with decreased survival in ovarian cancer [17–19]. On the other side, granulocytes have largely been neglected by tumor immunologists [20]. Challenging this, recent studies implied that neutrophil granulocytes might play an important role in the prevention of cancer metastasis [21]. Finally, neutrophil granulocytes are thought to have the capacity to undergo differentiation into N1 and N2 cells with anti- and pro-tumor properties, respectively [22, 23]. Therefore tumor infiltrating granulocytes regain attention in research [24–26].

In a previous study we could demonstrate that IL-17, but not FOXP3 positive immune cell infiltration in primary and recurrent ovarian carcinoma were indicative of chemosensitivity [27]. Finally it has been shown that IL-17 can be produced by granulocytes [28, 29] and other innate immune cells [30] and not only by TILs. However, in ovarian cancer the role of the innate immune system has not been evaluated to the same extent as the role of the adaptive immune system. In early stage lung cancer granulocytes have recently been shown to stimulate T cell responses in humans [31].

Neutrophilic granulocytes (NG) accumulate myeloperoxidase (MPO) in high amounts during their early maturation phase [32]. MPO produces hypochlorous acid from hydrogen peroxide and chloride anion and is responsible for the oxidization of tyrosine to tyrosyl radicals. Both are cytotoxic to a variety of microorganisms. After activation of granulocytes this enzyme is also implicated in the induction of apoptosis [33, 34].

There are few studies reporting the prognostic and predictive role of MPO in ovarian cancer. Therefore, we investigated its predictive value for chemosensitivity alone and in combination with IL-17 expression in a well characterized cohort of primary ovarian carcinomas and their matched recurrences which has also been used for previous publications of our group [35–38].

## Methods

### Patients

Tissues from ovarian serous carcinomas and their recurrences were available at the Pathology Biobank at Pathology of the University Hospital of Basel and the Cantonal Hospitals of Baden, Liestal and St. Gallen, Switzerland.

Mostly high-grade ovarian carcinomas (5.7 % FIGO stage II, 84.3 % FIGO stage III and 4.3 % FIGO stage IV) were included in this study after typing according to previous publications [39, 40]. The tissue microarray (TMA) was available from previous studies [35–38]. All patients had recurrences after initial surgery and had received at least three cycles of platinum-based adjuvant chemotherapy. The collection was divided into two groups according to response to chemotherapy. Recurrence occurring within 6 months after completion of platinum-based chemotherapy was defined as resistance [41]. The TMA allows investigation of tissues from ovarian carcinomas and matched recurrences from the same patients as previously shown [35–38]. The statement concerning the clinical data collection and ethical considerations can be found in previous publications [27, 35–38].

### Tissue microarray construction

The construction of the tissue microarray has been previously described [27, 42].

### Immunohistochemistry (IHC) and visual analysis

**Fig. 1:**
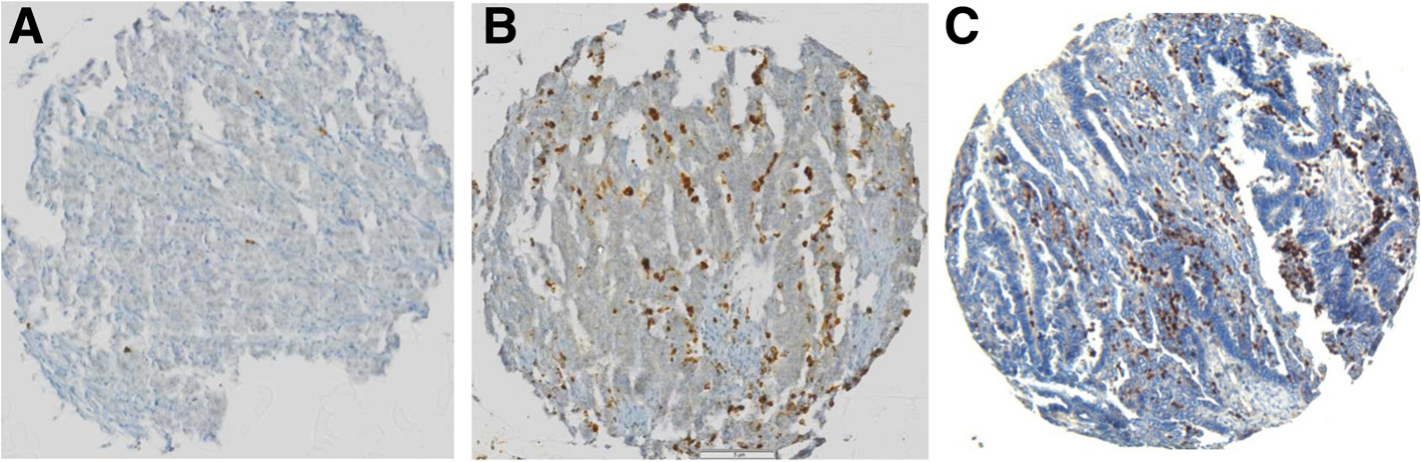
MPO and corresponding IL-17 specific staining in high grade ovarian carcinoma. Tumor punches are representative of low (panel **a**) and high (panel **b**) density of MPO positive TICI. Panel **c** shows an IL-17 specific staining in a section from the same biopsy shown in **b**. Magnification: 10×

Standard indirect immunoperoxidase procedures (ABC-Elite, Vectra Laboratories) were used for immunohistochemistry. For MPO staining the following antibody was used: clone 59A5 Novocastra, Newcastle, UK. In each tissue spot positive stained tumor immune cell infiltration (TICI) in the stroma was counted, representing approximately one high-power-field (10×), intravascular cells were excluded from analysis (Fig. 1a and b). Two independent experienced observers (RM and GS) analyzed the staining for specificity and the amount of TICI as described above. Cut-off was 22 positive cells/punch for MPO. Conclusive data for MPO were available in 47 biopsies of primary and 45 biopsies of matched recurrent carcinomas, respectively.

### Statistical analysis section

Cut-off scores used to classify ovarian carcinomas with low or high MPO infiltration were obtained by regression tree analysis, evaluating the best threshold in order to predict patients’ survival status, on all tumor samples [43]. Specific scores were set at 22 positive cells/punch. IL-17 data were available from a previous publication [27]. Kruskal Wallis, Chi-Square or Fisher’s Exact tests were used for the association of the clinicopathological features with the corresponding four groups of the biomarkers. Univariate recurrence-free and overall survival analysis was carried out by the Kaplan-Meier method and log rank test.

The assumption of proportional hazards was verified for both markers by analyzing the correlation of Schoenfeld residuals and the ranks of individual failure times. Any missing clinicopathological information was assumed to be missing at random. Subsequently, a multivariate Cox regression analysis was performed including MPO and IL-17. The hazard ratios (HR) and the 95 % confidence intervals (CI) were used to determine prognostic effects on survival time. Spearman’s rank correlation was used to analyze the correlation between MPO and IL-17. All statistical analyses were made using STATA software version 13 (StataCorp, College Station, TX, USA).

## Results

### Patient characteristics

The baseline characteristics of the patient cohort have been described previously [27] (Table 1). Briefly recurrence-free (RFS) and overall survival (OS) in the chemoresistant group was significantly shorter than in the chemosensitive group (2.2 ± 0.3 vs 18.2 ± 2.0 months, *p* < 0.0001 and 27 ± 5.3 vs. 49.6 ± 4.0 months, *p* = 0.0003, respectively). The analysis by MPO density is summarized in Table 2.

**Table 1:** Patient characteristics (*n* = 47)^a^

	*n* (%)
Age (median, range)	58 (34–77)
FIGO stage	
II	1 (2.1)
IIIA	1 (2.1)
IIIB	5 (10.6)
IIIC	32 (68.2)
IV	8 (17.0)
Residual disease	
None	16 (34.0)
<2 cm	17 (36.2)
>2 cm	13 (27.7)
Numbers of chemotherapy cycles	
<6	7 (14.9)
6 or more	39 (83.0)
CS^b^	33 (70.2)
CR^b^	14 (29.8)
RFS^c^ (mean/SE)	10.1 (1.4)
OS^c^ (mean/SE)	41.4 (4.3)

^a^missing clinicopathological information was assumed to be missing at random

^b^ *CS* chemosensitive, *CR* chemoresistant

^c^ *RFS* recurrence-free survival, *OS* overall survival

**Table 2:** Patients’ characteristics according to dichotomized distribution of MPO in the overall cohort (*n* = 47)^a^

	MPO high	MPO low	*p*-value
	*n* = 12 (100 %)	*n* = 35 (100 %)	
Age (median, range)	55 (45–73)	60 (34–77)	0.196
FIGO stage			
II	0	1 (2.9)	0.401
IIIA	0	1 (2.9)	
IIIB	3 (25.0)	2 (5.7)	
IIIC	7 (58.3)	25 (71.4)	
IV	2 (16.7)	6 (17.1)	
Residual disease			
None	4 (33.3)	12 (34.3)	0.919
<2 cm	5 (41.7)	12 (34.3)	
>2 cm	3 (25.0)	10 (28.6)	
Numbers of chemotherapy cycles			
<6	1 (8.3)	6 (17.1)	0.440
6 or more	11 (91.7)	28 (80.0)	
Primary cancer biopsies			
CS^b^	10 (83.3)	23 (65.7)	0.249
CR^b^	2 (16.7)	12 (34.3)	
Recurrent cancer biopsies (*n* = 10/35)			
CS^b^	9 (90.0)	23 (65.7)	0.135
CR^b^	1 (10.0)	12 (34.3)	
RFS^c^ (mean/SE)	11.9 (2.7)	9.5 (1.6)	0.380
OS^c^ (mean/SE)	56.8 (14.4)	39.3 (4.4)	0.139

^a^percentages may not add to 100 % due to missing values of defined variables, missing clinicopathological information was assumed to be missing at random. Variables are indicated as absolute numbers, %, median or range. Age, RFS and OS were evaluated using the Kruskal-Wallis test. FIGO stage, residual disease, numbers of chemotherapy cycles and chemoresistance were analyzed using the Chi-Square or the Fisher’s Exact test

^b^ *CS* chemosensitive, *CR* chemoresistant

^c^ *RFS* recurrence-free survival, *OS* overall survival

### MPO positive immune cell infiltration in paired primary and recurrent ovarian carcinoma

Mean number of infiltrating MPO positive cells in primary and recurrent cancer biopsies were 16.6 (±21.6) and 19.0 (±34.8), respectively. Neither for dichotomized MPO density in primary, nor in recurrent cancer biopsies a significant association with any clinicopathological feature was found (Table 2). Twelve out of 47 and 10 out of 45 displayed a high MPO cell density in primary and recurrent cancer biopsies, respectively. Finally, MPO density in primary and recurrent cancer biopsies did not show any significant association with chemosensitivity (*p* = 0.249 and *p* = 0.135) or any other clinicopathological feature (Table 2).

### Correlation analysis of MPO and IL-17 positive tumor immune cell infiltration

For more information concerning the relationship of MPO and IL-17 positive cell infiltration a correlation analysis of both markers was performed. Interestingly MPO and IL-17 positive TICI correlated significantly in all biopsies (*r*_s_ = 0.42; *p* < 0.001), in biopsies of only primary (*r*_s_ = 0.41; *p* = 0.004) and in biopsies of only recurrent carcinomas (*r*_s_ = 0.40; *p* = 0.007).

### Combined analysis of MPO and IL-17 positive cell density

**Fig. 2:**
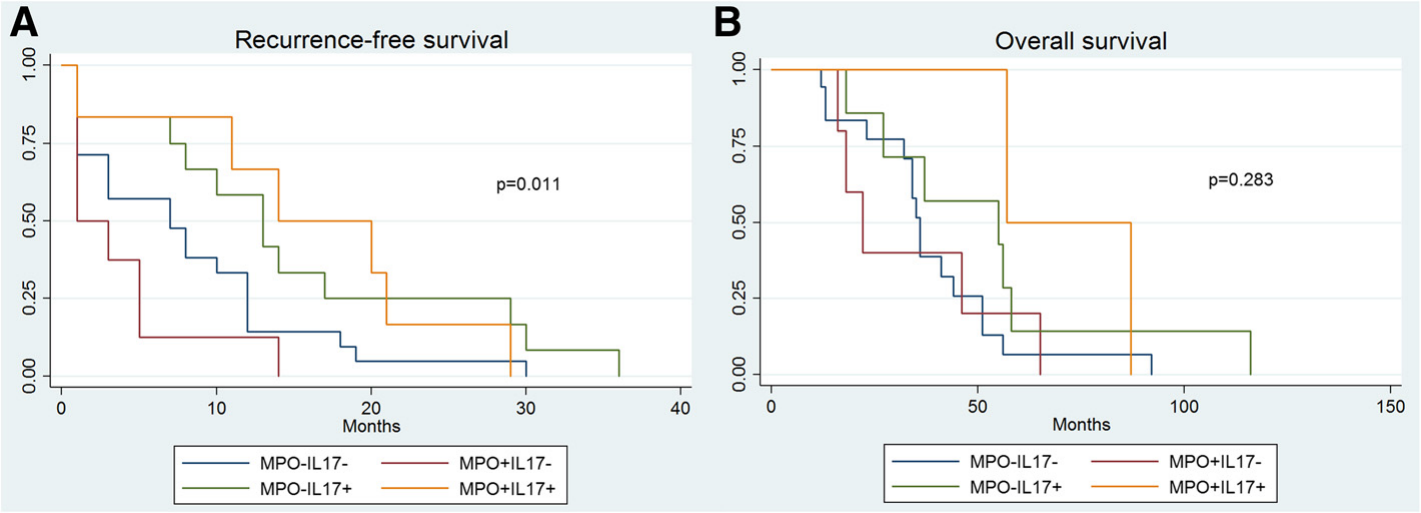
**a** Kaplan Meier survival curve of recurrence-free survival according to MPO and IL-17 density in primary cancer biopsies. Impact of MPO+ and IL-17+ tumor infiltrating immune cells on recurrence-free survival in patients with high grade ovarian carcinoma. Kaplan-Meier recurrence-free survival curves were split according to MPO+ and IL-17+ cell density in patients bearing high grade ovarian carcinoma as indicated. Cut-off values established by regression tree analysis were 22 cells/punch for MPO and 1 cell/punch for IL-17 cell infiltration. Cumulative effects of tumor infiltration by MPO+ and IL-17+ cells were explored. Blue line indicates to tumors with low MPO+ and low IL-17+ cell infiltration. Green line refers to tumors with high IL-17+ cell infiltration. Red line refers to tumors with high MPO+ cell infiltration and yellow line refers to tumors with high MPO+ and high IL-17+ cell infiltration. **b** Kaplan Meier survival curve of overall survival according to MPO and IL-17 density in primary cancer biopsies. Impact of MPO+ and IL-17+ tumor infiltrating immune cells on overall survival in patients with high grade ovarian carcinoma. Kaplan-Meier overall survival curves were split according to MPO+ and IL-17+ cell density in patients bearing high grade ovarian carcinoma as indicated. Cut-off values established by regression tree analysis were 22 cells/punch for MPO and 1 cell/punch for IL-17 cell infiltration. Cumulative effects of tumor infiltration by MPO+ and IL-17+ cells were explored. Blue line indicates to tumors with low MPO+ and low IL-17+ cell infiltration. Green line refers to tumors with high IL-17+ cell infiltration. Red line refers to tumors with high MPO+ cell infiltration and yellow line refers to tumors with high MPO+ and high IL-17+ cell infiltration

Based on the correlation analysis results, a combined analysis of MPO and IL-17 cell density was performed. As shown in Fig. 2a, MPO and IL-17 positive TICI frequency was significantly associated with a longer RFS in biopsies of primary cancers (*n* = 47, *p* = 0.011), although the combined marker analysis did not show significant association with OS (Fig. 2b, *p* = 0.283). In addition, there was a significant association with chemosensitivity (*p* = 0.004) and FIGO classification (*p* = 0.029) in primary cancer biopsies (Tables 3 and 4).

**Table 3:** Dichotomized distribution of MPO and IL-17 according to defined cut-offs (22 cells/punch for MPO and 1 cell/punch for IL-17 [27] in primary carcinomas (*n* = 47)^a^

	MPO-/IL17- *n* = 21 (100 %)	MPO+/IL17- *n* = 8 (100 %)	MPO-/IL17+ *n* = 12 (100 %)	MPO+/IL17+ *n* = 6 (100 %)	*p*-value
Age (median, range)	62 (39–77)	59.5 (50–65)	57 (34–72)	55 (45–73)	0.742
FIGO stage					
II	0	0	1 (8.3)	0	**0.029**
IIIA	1 (4.7)	0	0	0	
IIIB	2 (9.5)	0	0	3 (50.0)	
IIIC	17 (81.1)	5 (62.5)	8 (66.7)	2 (33.3)	
IV	1 (4.7)	3 (37.5)	3 (25.0)	1 (16.7)	
Residual disease					
None	9 (42.9)	2 (25.0)	3 (25.0)	2 (33.3)	0.262
<2 cm	6 (28.6)	1 (12.5)	7 (58.3)	3 (50.0)	
>2 cm	5 (23.8)	5 (62.5)	2 (16.7)	1 (16.7)	
Numbers of chemotherapy cycles					
<6	5 (23.8)	0	1 (8.3)	1 (16.7)	0.412
6 or more	15 (71.4)	8 (100.0)	11 (91.7)	5 (83.3)	
CS^b^	14 (66.7)	2 (25.0)	11 (91.7)	6 (100.0)	**0.004**
CR^b^	7 (33.3)	6 (75.0)	1 (8.3)	0	
RFS^c^ (mean/SE)	8.1 (1.7)	3.9 (1.6)	14.9 (3.3)	16 (3.9)	**0.024**
OS^c^ (mean/SE)	36 (4.5)	33.4 (9.6)	52.4 (12.1)	72 (15.0)	0.113

Bold data statistically significant *p* < 0.05

^a^percentages may not add to 100 % due to missing values of defined variables, missing clinicopathological information was assumed to be missing at random. Variables are indicated as absolute numbers, %, median or range. Age, RFS and OS were evaluated using the Kruskal-Wallis test. FIGO stage, residual disease, numbers of chemotherapy cycles and chemoresistance were analyzed using the Fisher’s Exact test

^b^ *CS* chemosensitive, *CR* chemoresistant

^c^ *RFS* recurrence-free survival, *OS* overall survival

**Table 4:** Dichotomized distribution of MPO and IL-17 according to defined cut-offs (22 cells/punch for MPO and 1 cell/punch for IL-17 [27] in recurrent carcinomas (*n* = 45)^a^

	MPO-/IL17- *n* = 23 (100 %)	MPO+/IL17- *n* = 4 (100 %)	MPO-/IL17+ *n* = 12 (100 %)	MPO+/IL17+ *n* = 6 (100 %)	*p*-value
Age (median, range)	62 (47–77)	59.5 (45–69)	59 (34–68)	54.5 (39–60)	0.277
FIGO stage					
II	0	0	1 (8.3)	0	0.574
IIIA	0	0	1 (8.3)	0	
IIIB	2 (8.7)	0	2 (16.7)	1 (16.7)	
IIIC	17 (73.9)	4 (100.0)	6 (50.0)	3 (50.0)	
IV	4 (17.4)	0	2 (16.7)	2 (33.3)	
Residual disease					
None	7 (30.4)	0	6 (50.0)	2 (33.3)	**0.046**
<2 cm	5 (21.7)	3 (66.7)	5 (41.7)	4 (66.7)	
>2 cm	10 (43.5)	1 (33.3)	1 (8.3)	0	
Numbers of chemotherapy cycles					
<6	3 (13.0)	0	3 (25.0)	1 (16.7)	0.806
6 or more	19 (82.6)	4 (100.0)	9 (75.0)	5 (83.3)	
CS^b^	13 (56.5)	3 (66.7)	10 (83.3)	6 (100.0)	0.123
CR^b^	10 (43.5)	1 (33.3)	2 (16.7)	0	
RFS^c^ (mean/SE)	7.7 (1.8)	12.5 (7.1)	14.2 (2.5)	8.3 (2.2)	0.121
OS^c^ (mean/SE)	33.5 (5.8)	20 (2.0)	53.4 (9.3)	45.2 (6.5)	0.074

Bold data statistically significant *p* < 0.05

^a^percentages may not add to 100 % due to missing values of defined variables, missing clinicopathological information was assumed to be missing at random. Variables are indicated as absolute numbers, %, median or range. Age, RFS and OS were evaluated using the Kruskal-Wallis test. FIGO stage, residual disease, numbers of chemotherapy cycles and chemoresistance were analyzed using the Fisher’s Exact test

^b^ *CS* chemosensitive, *CR* chemoresistant

^c^ *RFS* recurrence-free survival, *OS* overall survival

In biopsies from carcinoma recurrences, no significant association with RFS could be found (*p* = 0.121).

### Multivariate analysis of synergistic effect

In a multivariate cox regression analysis including age, residual disease, FIGO classification, number of chemotherapy cycles and categorized MPO and IL-17 cell density, the combination of the immune markers was an independent prognostic factor for RFS in primary cancer biopsies (Table 5).

**Table 5:** Multivariate Hazard Cox regression analysis of recurrence-free survival considering the categorized combination of both markers

	HR	95 % CI	*p*-values
Age	0.99	0.96–1.02	0.612
MPO + IL17-	0.64	0.17–2.48	0.518
MPO-IL17+	0.26	0.10–0.69	**0.006**
MPO + IL17+	0.23	0.07–0.73	**0.013**
Residual disease <2 cm	1.03	0.48–2.20	0.949
Residual disease >2 cm	3.93	1.47–10.52	**0.007**
N of chemotherapy cycles	1.16	0.89–1.52	0.276
FIGO IIIA	0.08	0.00–1.88	0.115
FIGO IIIB	0.58	0.05–6.48	0.661
FIGO IIIC	0.40	0.04–3.61	0.413
FIGO IV	0.59	0.06–6.00	0.652

Bold data statistically significant *p* < 0.05

Multivariate analyses showing Hazard Ratios and *p*-value for all primary cancer biopsies (*n* = 46 less than 47 due to missing values) conferred by categorized MPO/IL-17 density, age, residual disease after cytoreductive surgery, number of chemotherapy cycles and FIGO classification

## Discussion

As in ovarian carcinoma, surgical tumor debulking is often followed by adjuvant platinum-based chemotherapy it would be helpful to find predictive markers for chemoresponse. Based on such biomarkers it would be possible to carry out extended chemotherapy regimen and repetitive surgical procedures. In our previous studies we identified different potential therapeutical targets and IL-17 as predictive marker for chemosensitivity [27, 35–38]. As previously mentioned there is a resurgent interest in the scientific community concerning the role of granulocytes in tumor immunology [22, 24, 31]. Indeed, they have usually been associated with poor prognosis [20]. But experimental models in the past have proposed an anti-tumor role through the activation of T cells [44, 45]. Furthermore recently, granulocyte polarization has been described [22] and the ability of granulocytes to promote lymphocyte activation in the tumor environment has been reported [31].

The composition of tumor microenvironment has been shown to significantly impact tumor progression and clinical outcome [12]. Similar to a variety of cancers of different origins, T lymphocyte infiltration in ovarian cancer has a positive prognostic role [46]. Most interestingly, ovarian cancer infiltration by IL-17 producing cells has been found to be associated with either longer RFS or good prognosis by us and others [27, 47]. However, there is no evidence concerning the potential clinical relevance of granulocyte infiltration in ovarian cancer.

In our previous study we found that IL-17 positive TICI were significantly more frequent in the chemosensitive ovarian carcinoma group [27]. Granulocytes and other immune cells of the innate immune system can produce IL-17 [28–30]. On the other hand, TNF-alpha in the tumor microenvironment could attract myeloid cells in an IL-17-dependent manner and contribute to tumor-promotion [48]. Therefore we investigated the predictive role of MPO, an enzyme that is expressed in myeloid cells, alone and together with IL-17. Finally we found that the combination of both markers was an independent prognostic factor for RFS. However, in this study IL-17 was the dominant marker for prediction of RFS. A further limitation is the small sample size. Therefore, our results have to be validated in an independent larger patient cohort.

## Conclusion

Based on the results in this study, we conclude that the combination of high MPO positive cell density and IL-17 expression enhances the indicative value for the response of ovarian carcinomas to chemotherapy, as it in addition has prognostic value regarding recurrence-free survival in ovarian carcinoma. Although, these results have to be validated in a larger cohort.

## Abbreviations

IL-17: Interleukin-17
MPO: Myeloperoxidase
TMA: Tissue microarray
